# USING INFORMATICS AND GENERATIVE AI TO SUPPORT OLDER ADULTS’ UNDERSTANDING OF LAB TEST RESULTS

**DOI:** 10.1093/geroni/igae098.0351

**Published:** 2024-12-31

**Authors:** Zhe He, Balu Bhasuran, Qiao Jin, Shubo Tian, Karim Hanna, Zhiyong Lu

**Affiliations:** Florida State University, Tallahassee, Florida, United States; Florida State University, Tallahassee, Florida, United States; National Center for Biotechnology Information (NCBI), National Library of Medicine (NLM), National Institutes of Health, Bethesda, Maryland, United States; National Center for Biotechnology Information (NCBI), National Library of Medicine (NLM), National Institutes of Health, Bethesda, Maryland, United States; University of South Florida, Tampa, Florida, United States; National Center for Biotechnology Information (NCBI), National Library of Medicine (NLM), National Institutes of Health, Bethesda, Maryland, United States

## Abstract

Viewing laboratory test results is the most frequently used function of patient portals, but lab results can be confusing for patients. In this project, we aim to gain insights into older adults’ challenges in understanding lab test results. To achieve the goals, we first conducted an online survey with 276 patients with one or more chronic conditions. We found that younger adults and patients who had never used patient portals had significantly lower lab test comprehension scores compared to older adults, and those with higher numeracy or with no multiple chronic conditions. Even though older adults performed better on lab test comprehension tests, most reported they have difficulty understanding lab results online and prefer that their doctors explain these to them. In the second study, we selected 53 lab results-related questions from a community Q&A website, Yahoo! Answers, and evaluated the answers to these questions from four generative large language models. We utilized a ChatGPT-based evaluator Win Rate to judge whether a target model has higher quality in terms of relevance, correctness, helpfulness, and safety than the baseline model. Then, we performed a manual evaluation with medical experts for all the responses to seven selected questions on the same four aspects. The results of Win Rate and medical expert evaluation both showed that GPT-4’s responses achieved better scores than all the other LLM responses from LLaMa 2, ORCA_mini, and MedAlpaca and human responses on all four aspects. However, LLM responses occasionally suffer from incorrect statements.

